# How Biofilm Growth Affects *Candida*-Host Interactions

**DOI:** 10.3389/fmicb.2020.01437

**Published:** 2020-06-25

**Authors:** Emily F. Eix, Jeniel E. Nett

**Affiliations:** Departments of Medicine and Medical Microbiology and Immunology, University of Wisconsin-Madison, Madison, WI, United States

**Keywords:** *Candida*, biofilm, host, neutrophil, macrophage, matrix, immunity, dispersion

## Abstract

*Candida* spp. proliferate as surface-associated biofilms in a variety of clinical niches. These biofilms can be extremely difficult to eradicate in healthcare settings. Cells within biofilm communities grow as aggregates and produce a protective extracellular matrix, properties that impact the ability of the host to respond to infection. Cells that disperse from biofilms display a phenotype of enhanced pathogenicity. In this review, we highlight host-biofilm interactions for *Candida*, focusing on how biofilm formation influences innate immune responses.

## Introduction

*Candida* spp. are the primary cause of nosocomial fungal infections and recently rose to the leading pathogen group causing nosocomial bloodstream infections (Magill et al., 2014). *Candida* spp. exhibit the propensity to proliferate as adherent biofilms (Magill et al., 2014; Nobile and Johnson, 2015). These aggregated communities exhibit resistance to antifungals as well as host immune responses, making them extremely difficult to eradicate (Chandra et al., 2001a; Donlan,2001b; Douglas, 2003). In the hospital setting, *Candida* spp. form biofilms on artificial medical devices, such as vascular catheters, which can lead to bloodstream infection and disseminated disease with associated mortality of approximately 30% (Donlan,2001a; Kojic and Darouiche, 2004; Wisplinghoff et al., 2004; Kumamoto and Vinces, 2005; Pfaller and Diekema, 2007; Tumbarello et al., 2007). It is estimated that nearly 80% of patients with invasive candidiasis have implanted medical devices (Andes et al., 2012). The ability of *Candida* spp. to persist as a biofilm on these devices poses a serious issue for treatment of *Candida* infections, as device removal is often the only option (Pfaller and Diekema, 2007; Andes et al., 2012). However, even with catheter removal, mortality rates remain high in the setting of invasive candidiasis (Andes et al., 2012). The observation that removal of catheters decreases the risk of persistent candidemia and rate of mortality suggests that biofilm formation plays a major role in the pathogenesis of invasive candidiasis (Andes et al., 2012; Ala-Houhala and Anttila, 2020).

**FIGURE 1:**
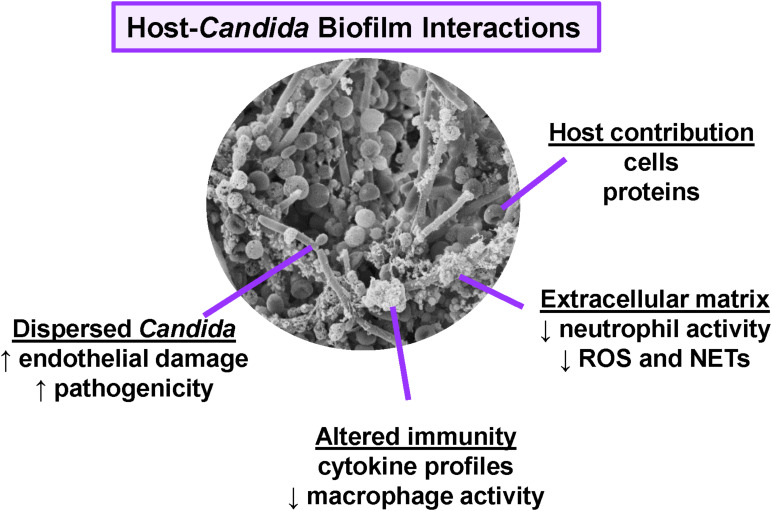
Overview of host interactions with *Candida* biofilms. Scanning electron micrograph reveals *C. albicans* (SC5314) growing as a biofilm on the luminal surface of a rat vascular catheter. Biofilm formation influences host interactions, immunity, and pathogenesis.

*Candida albicans*, the most prevalent *Candida* spp., has served as a model organism for study of biofilm formation (Hawser and Douglas, 1994; Chandra et al., 2001b; Ramage et al., 2001; Uppuluri et al., 2010). However, biofilm formation is not unique to *C. albicans*, as many other clinically relevant *Candida* spp., including *C. glabrata, C. tropicalis, C. parapsilosis*, and the emerging pathogen *C. auris*, also form biofilms (Kuhn et al., 2002; Lewis et al., 2002; Jain et al., 2007; Bizerra et al., 2008; Sherry et al., 2017). While biofilms formed by different *Candida* spp. may vary in morphology and density, the structures uniformly contain a polymeric extracellular matrix that encases and protects the fungal cells. The components of the extracellular matrix differ from those found in the *Candida* cell wall, and these moieties are proposed to modulate host recognition by concealing the cell wall components that typically interact with the immune system (Johnson et al., 2016; Zawrotniak et al., 2017; Hoyer et al., 2018). In addition, cells dispersed from biofilms exhibit characteristics distinct from cells growing under non-biofilm conditions (Sellam et al., 2009; Uppuluri et al., 2010). In this review, we highlight key host interactions with *Candida* biofilms, describing how the host responds differently to *Candida* during biofilm and non-biofilm growth (Figure 1).

## Impact Of Biofilm Formation On Neutrophil Responses

Neutrophils serve as primary innate immune responders to *Candida* and are critical for controlling invasive infection (FidelJr.,2002). The susceptibility of neutropenic patients to severe fungal infections, including candidiasis, highlights the importance of these leukocytes (EdwardsJr., Lehrer et al., 1978; Erwig and Gow, 2016). However, when growing as a biofilm, *Candida* resists damage and killing by neutrophils (Katragkou et al., 2010, 2011a,b; Xie et al., 2012; Johnson et al., 2016; Kernien et al., 2017). Compared to the neutrophil response to planktonic *C. albicans*, neutrophils exhibit an up to 5-fold lower activity against biofilms formed by *C. albicans* (Katragkou et al., 2010, 2011b; Xie et al., 2012; Johnson et al., 2016). This process appears to involve induction of an inhibitory pathway, as neutrophils exposed to *C. albicans* resist activation by potent stimuli, such as phorbol ester. Additionally, priming of neutrophils with pro-inflammatory cytokines, including interferon-γ and granulocyte colony-stimulating factor, does not restore neutrophil killing of biofilms (Katragkou et al., 2011b). This inhibitory process appears independent of filamentation, as *C. albicans* biofilms of various architectures, including those comprised primarily of yeast morphotypes, similarly impair neutrophil function. Consistent with this, biofilms formed by *C. glabrata* and *C. parapsilosis*, which lack true hyphae, also resist neutrophil attack (Katragkou et al., 2011a; Johnson et al., 2017).

Neutrophils respond to pathogens via a variety of effector mechanisms, including phagocytosis, production of reactive oxygen species (ROS), and the formation of neutrophil extracellular traps (NETs), which are web-like structures containing DNA, histones, and antimicrobial proteins (Brinkmann et al., 2004). While neutrophils are capable of phagocytosing *Candida* yeast cells, the elongated hyphal cells cannot be completely engulfed. Instead, in response to *C. albicans* hyphae and other large or aggregated pathogens, neutrophils release NETs (Urban et al., 2006; Branzk et al., 2014). This process of NET formation would arguably be an effective neutrophil response against biofilm, considering their large size and the inability of the structures to be completely phagocytosed. However, NETs are not produced in response to *C. albicans* biofilms (Johnson et al., 2016; Kernien et al., 2017). *C. albicans* biofilms do not trigger neutrophils to generate ROS, a signaling pathway that governs many forms of NET formation (Brinkmann et al., 2004; Xie et al., 2012; Johnson et al., 2016). These impaired responses have been attributed, in part, to the presence of an extracellular matrix encasing the cells (Johnson et al., 2016).

## Monocyte and Macrophage Responses to Biofilm

The formation of biofilm influences a variety of mononuclear innate immune cell responses, including migration, phagocytosis, and cytokine production (Chandra et al., 2007; Katragkou et al., 2010; Alonso et al., 2017; Simitsopoulou et al., 2018; Arce Miranda et al., 2019). In addition, the presence of these and other cells can impact biofilm formation by *Candida*. For example, when incubated with *C. albicans* biofilm, peripheral blood mononuclear cells fail to engage in phagocytosis (Chandra et al., 2007). However, the presence of these cells promotes the formation of a thicker, hyphal-rich biofilm, which has been linked to a biofilm-enhancing soluble factor produced by the mononuclear cells during co-culture with biofilm (Chandra et al., 2007).

In addition to avoiding phagocytosis, mononuclear cells exposed to biofilms also display an altered cytokine profile, when compared to those interacting with planktonic *C. albicans* (Chandra et al., 2007). Observed differences for biofilm exposure include increases in both pro-inflammatory cytokines (IL-1β and MCP-1), as well as the anti-inflammatory cytokine, IL-10 (Chandra et al., 2007). Additional studies utilizing a human monocytic cell line (THP-1) have also shown differing cytokine responses to biofilm and planktonic *C. albicans*, including lower TNF-α production upon biofilm exposure (Katragkou et al., 2010). Modulation of cytokine production by biofilm likely influences immunity, but little is known about this process. However, it appears that cytokine responses to *Candida* biofilm may vary among species (Simitsopoulou et al., 2018).

Investigations using murine macrophage cell lines are beginning to shed light on the impact of *Candida* biofilm formation on macrophage interactions (Alonso et al., 2017; Arce Miranda et al., 2019). During the initiation of biofilm formation, macrophages are capable of phagocytosing *C. albicans* (Arce Miranda et al., 2019). However, as biofilms mature, macrophages do not exhibit activity against them, and may even enhance biofilm production (Arce Miranda et al., 2019). This pattern of impaired activity against mature biofilms is similar to that observed for both human mononuclear cells and neutrophils (Chandra et al., 2007; Katragkou et al., 2010, 2011a,b; Xie et al., 2012; Johnson et al., 2016; Kernien et al., 2017). Like the neutrophil response to *C. albicans* biofilms, macrophage-biofilm interactions also involve diminished ROS production (Xie et al., 2012; Johnson et al., 2016; Arce Miranda et al., 2019). Another response impaired by *C. albicans* biofilms is macrophage migration. Murine macrophages move at rates approximately 2-fold lower in response to biofilm when compared to incubation with planktonic *C. albicans* (Alonso et al., 2017).

## Role of *Candida* Biofilm Matrix in Immunity

The development of *Candida* biofilm begins with adherence to a substrate, which is followed by proliferation and the assembly of an extracellular matrix, a hallmark characteristic of mature biofilm formation (Chandra et al., 2001a; Uppuluri et al., 2010; Wall et al., 2019). This extracellular matrix encases the cells and presents unique structures which conceal the cell wall components that are typically encountered by innate immune cells (Chaffin et al., 1998; Hawser et al., 1998). Many studies have demonstrated altered immune cell interactions with *Candida* biofilms (Chandra et al., 2007; Katragkou et al., 2011a; Kernien et al., 2017; Xie et al., 2012; Johnson et al., 2016, 2017; Alonso et al., 2017; Arce Miranda et al., 2019). For neutrophils, this phenotype appears to require an intact extracellular matrix, as disruption of biofilm matrix can restore neutrophil activity, including their ability to produce NETs and damage biofilms (Katragkou et al., 2010; Johnson et al., 2016). It is likely that extracellular matrix contributes to other aspects of immune modulation by biofilms as well.

Analysis of *C. albicans* biofilms demonstrates that extracellular matrix contains a mixture of biopolymers, including proteins (55%), carbohydrates (25%), lipids (15%), and nucleic acids (5%) (Zarnowski et al., 2014). Many of these components differ from those found in the cell wall. For example, the biofilm extracellular matrix of *C. albicans* contains an abundant high molecular weight α-1,2-branched α-1,6 mannan, which assembles with linear β-1,6 glucan to form a mannan-glucan complex that is not found in the cell wall (Chaffin et al., 1998; Zarnowski et al., 2014; Mitchell et al., 2015). This complex has been linked to the capacity of *C. albicans* biofilms to inhibit neutrophil function, as *C. albicans* biofilms with genetic disruption of this pathway activate neutrophils (Johnson et al., 2016). Neutrophils then generate ROS and produce NETs in response to these mutants lacking extracellular matrix, ultimately resulting in fungal damage (Johnson et al., 2016). The fungal components involved in triggering this response are not certain. The finding that neutrophils are also activated by biofilms following treatment with echinocandin drugs, which unmask β-1,3 glucan, suggests a role for this polysaccharide (Katragkou et al., 2010; Hoyer et al., 2018; Simitsopoulou et al., 2018).

The production of matrix mannan-glucan complex is conserved across *Candida* species, including *C. albicans, C. tropicalis, C. parapsilosis, C. glabrata*, and *C. auris* (Dominguez et al., 2018, 2019). It is anticipated that similar mechanisms of neutrophil evasion may occur upon encounter with biofilms formed by these species as well. However, species-specific immune responses have been observed (Simitsopoulou et al., 2018). Additional study is needed to delineate the neutrophil-biofilm interactions for these and other emerging species.

## Host Response to Dispersed Biofilms

Throughout biofilm development, cells detach from biofilms, allowing *Candida* to disseminate to the bloodstream and cause invasive disease (Uppuluri et al., 2010). Various environmental responses, including carbon source and pH, trigger this regulated process for *C. albicans* (Sellam et al., 2009; Uppuluri et al., 2010). During dispersion, yeast-like cells bud from the upper biofilm layer of hyphae and are release as elongated cells (Uppuluri et al., 2010). Although the cells resemble yeast, the newly dispersed cells display enhanced pathogenicity traits, including heightened capacities for filamentation, adhesion, and biofilm formation (Uppuluri et al., 2010). Even more striking, the dispersed cells exhibit enhanced virulence in a murine model of invasive candidiasis and exert more damage to endothelial cells (Uppuluri et al., 2010). The transcriptional profile of dispersed cells broadly differs from both biofilm and planktonic *C. albicans*, consistent with their unique phenotype (Sellam et al., 2009; Uppuluri et al., 2018). These cells likely play a major role in the pathogenesis of vascular catheter and other device-associated infections that result in disseminated disease. In this setting, environmental cues can trigger the detachment of yeast-form cells with a heightened propensity to disseminate though the bloodstream, adhere to endothelial cells, and damage tissues (Uppuluri et al., 2010).

## Insight Into Host Responses to Biofilm Through Animal Models

*Candida* spp. interact extensively with the host during infection, and animal models are ideal for examining this interface *in vivo*. Invasive candidiasis frequently involves biofilm proliferation on indwelling vascular catheters, which can lead to catheter-associated bloodstream infection and disseminated disease (Kojic and Darouiche, 2004; Andes et al., 2012). Models in mice, rats, and rabbits mimicking vascular catheter-associated infection have shed light on biofilm-host interactions (Andes et al., 2004; Schinabeck et al., 2004; Lazzell et al., 2009). These models reveal differences between *in vitro* and *in vivo* biofilms, including the formation of a thicker biofilm matrix *in vivo.* Interestingly, *in vivo* biofilm models reveal that a striking number (>95%) of host-derived proteins incorporate into the extracellular matrix, indicating a major host contribution to *C. albicans* biofilms (Nett et al., 2015). Host proteins depositing in the biofilm matrix include matricellular proteins, and proteins indicating the presence of erythrocytes and leukocytes. Imaging of catheters similarly shows the incorporation of many host cells, including erythrocytes and neutrophils (Andes et al., 2004). These findings suggest that neutrophils recruit to *C. albicans* biofilms *in vivo*, but lack significant anti-biofilm activity.

Urinary catheter biofilm models have been developed in mice and rats, allowing the study of catheter-associated candiduria (Wang and Fries, 2011; Nett et al., 2014; Capote-Bonato et al., 2018). As seen with the vascular catheter models, on urinary catheters, *C. albicans* also forms thick biofilms with dense extracellular matrix (Wang and Fries, 2011; Nett et al., 2015). Biofilms in this environment also incorporate numerous host cells and proteins, which contribute to the extracellular matrix (Nett et al., 2015). *C. tropicalis* similarly forms biofilms on urinary catheter segments in mice (Capote-Bonato et al., 2018). It appears that host response to both *C. albicans* and *C. tropicalis* biofilms involves a degree of neutrophilic infiltration (Nett et al., 2015; Capote-Bonato et al., 2018). However, the biofilms persist despite this response.

In addition to the vascular and urinary placement of catheters, models have employed subcutaneous implantation of catheters or other devices in mice and rats to elucidate host interactions with *Candida* biofilms (Ricicova et al., 2010; Nieminen et al., 2014; Kucharikova et al., 2015). These models primarily involve the insertion of preformed biofilms that continue to propagate *in vivo*. Both *C. albicans* and *C. glabrata* proliferate as biofilms in this setting (Ricicova et al., 2010; Nieminen et al., 2014; Kucharikova et al., 2015). The subcutaneous *C. albicans* biofilms induce an infiltration of inflammatory cells consisting predominantly of neutrophils and macrophages (Nieminen et al., 2014). Consistent with this, tissue sections adjacent to subcutaneous biofilms show an inflammatory profile with an increased abundance of inflammatory mediators, including matrix metalloproteinases and myeloperoxidase (Nieminen et al., 2014). Similar to other models of *C. albicans* biofilm formation, the biofilms withstand this defense.

Oral biofilms represent one of the most common niches for *Candida* biofilm formation (Douglas, 2002). Rats have predominantly been utilized to study host-biofilm interactions, particularly for the analysis of dental devices and the associated denture stomatitis (Nett et al., 2010; Lee et al., 2011; Johnson et al., 2012; Tobouti et al., 2016; Sultan et al., 2019; Yano et al., 2019). In these models, *Candida* spp. adhere to the artificial devices, proliferating as a biofilm (Nett et al., 2010; Chen et al., 2011; Johnson et al., 2012; Tobouti et al., 2016; Sultan et al., 2019). Similar to other sites of infection, host materials become intertwined in the extracellular matrix of these *C. albicans* biofilms, with integration of salivary proteins and immune cells into the matrix (Nett et al., 2015). The mucosal response involves the recruitment of inflammatory cells to the palate mucosa and epithelial changes consistent with the histopathology of dental stomatitis seen clinically (Nett et al., 2010; Johnson et al., 2012; Tobouti et al., 2016; Sultan et al., 2019).

While the clinical relevance of *Candida* biofilm in the pathogenesis of vulvovaginal candidiasis is not well-understood, murine vaginitis models reveal that *C. albicans* forms biofilm on the vaginal mucosa (Harriott et al., 2010). These mucosal biofilms are characterized by the presence of an extracellular matrix surrounding yeast and hyphal cells, typical of *in vitro C. albicans* biofilms and biofilms formed at other sites of infection (Harriott et al., 2010). Clinically, vulvovaginal candidiasis is associated with a robust neutrophil response leading to acute inflammation. A similar acute inflammatory response is recapitulated in a murine model of *C. albicans* infection, which appears to be mediated by the release of S100 alarmins from epithelial cells (Yano et al., 2010, 2012). Interestingly, this likely represents a species-specific host interaction, as *C. glabrata* does not form biofilm or elicit a strong inflammatory response in this model (Nash et al., 2016). Further studies are required to fully elucidate the complex host response to *Candida* biofilms during vaginal infection.

## Host Responses to *Candida* in Mixed-Species Biofilms

Many studies have focused on the proliferation of *Candida* in a single-species biofilm, but *Candida* spp. also form polymicrobial biofilms in a variety of niches, including the oropharynx and skin. In these mixed-species biofilms, the immune response to one species may influence immunity to another organism. For example, *Staphylococcus aureus* has been shown to preferentially adhere to *C. albicans* hyphae and form mixed-species biofilms (Peters et al., 2010; Zago et al., 2015; de Carvalho Dias et al., 2017). In this setting *C. albicans* promotes the phagocytosis of *Staphylococcus aureus*, which can be carried by phagocytes from the oral cavity to the lymphatic system and cause invasive, disseminated disease in murine model of candidiasis (Allison et al., 2019). In addition, formation of a mixed biofilm triggers differential production of soluble factors and proteins that are anticipated to modulate immunity and enhance the virulence of both species (Peters et al., 2010; de Carvalho Dias et al., 2017).

Chronic wounds frequently become colonized by polymicrobial biofilms, and *Candida* spp. are increasingly recognized as major contributors to these infections (Kalan et al., 2016; Kalan and Grice, 2018). Not only are *Candida* spp. among the most frequently isolated fungal pathogens from diabetic foot ulcers, the presence of fungi in these wounds correlates with longer healing times (Kalan et al., 2016). *In vitro* wound biofilm models recapitulate the chronic wound environment and shed light on interactions among common colonizers, including *C. albicans*, *Pseudomonas aeruginosa*, and *Staphylococcus aureus* in this context (Townsend et al., 2016, 2017). In a three-dimensional wound biofilm model, combined antibiotic and antifungal treatments are most effective in eliminating polymicrobial biofilms, emphasizing the importance of considering fungal presence in chronic wounds (Townsend et al., 2017). While much of the host response to these chronic wound biofilms has not yet been elucidated, this represents an important topic for future studies.

In addition to forming polymicrobial biofilms with bacteria, *C.albicans* also establishes mixed-species biofilms with other *Candida* (Pathirana et al., 2019; Tati et al., 2016; Vipulanandan et al., 2018). One common clinical example of this is oropharyngeal candidiasis, which often involves multiple *Candida* species (Redding, 2001). In a mouse model of oropharyngeal candidiasis, colonization by *C. glabrata* requires the presence of *C. albicans* (Tati et al., 2016). Co-culture of the organisms lead to upregulation of *C. glabrata* cell surface proteins that allow for adhesion to *C. albicans* hyphae (Tati et al., 2016). Additional examples of species involved in mixed-species biofilms include *C. dubliniensis* and *C. tropicalis*, both of which appear to adhere to *C. albicans* and exhibit a growth benefit (Pathirana et al., 2019). When co-cultured together, these species form biofilms that achieve higher surface coverage. The influence of these altered biofilm structures on host responses remains unclear. However, the host interface for mixed biofilms may be quite distinct from that observed for either species alone.

## Conclusions and Future Directions

Candidiasis frequently involves the formation of surface-associated biofilms. These structures have a multifaceted interaction with the host. Compared to cells grown in free-floating conditions, *Candida* biofilms exhibit resistance to phagocytosis by neutrophils, monocytes, and macrophages. In addition, biofilm formation alters mononuclear cell cytokine profiles, broadly influencing immunity. Biofilms modulate immunity throughout various developmental stages. During mature biofilm formation, extracellular matrix contributes to resistance to host defenses. As fungal cells disperse, a more virulent phenotype results in enhanced pathogenesis.

Further understanding of the impact of biofilm formation on host immunity will be of interest. Many biofilm studies have explored immune cell interactions *ex vivo*. However, biofilm composition is highly impacted by *in vivo* conditions, and little is known about how the host contribution to biofilm may alter immune recognition. In addition, studies are just beginning to shed light on the complexity of immunity to mixed biofilms. Furthermore, it will fascinating to see how biofilm formation by emerging species, such as *C. auris*, influences host responses, as *C. albicans* has primarily been utilized as a model organism.

## Author Contributions

EE and JN wrote the manuscript. Both authors contributed to the article and approved the submitted version.

## Conflict of Interest

The authors declare that the research was conducted in the absence of any commercial or financial relationships that could be construed as a potential conflict of interest.
